# Accessory Hepatic Tissue Attached to the Gallbladder: An Incidental Intraoperative Finding

**DOI:** 10.7759/cureus.97873

**Published:** 2025-11-26

**Authors:** Mehmet Dinçay Yar, Şebnem Çimen, Şahin Kaymak, Ali Kağan Coşkun

**Affiliations:** 1 Department of General Surgery, Gülhane Training and Research Hospital, University of Health Sciences, Ankara, TUR; 2 Department of General Surgery, Akyurt State Hospital, Ankara, TUR

**Keywords:** accessory liver tissue, anatomic variations, ectopic liver tissue, hepatobiliary anomalies, malignant transformation

## Abstract

Ectopic liver tissue is a rare developmental anomaly that is usually asymptomatic and often detected incidentally during abdominal surgery. A 58-year-old female patient with a 13-year history of type 2 diabetes mellitus and chronic steatohepatitis presented with recurrent biliary colic. Preoperative ultrasonography demonstrated a 1 cm gallstone, hepatomegaly (long axis 183 mm), and grade 2 hepatic steatosis. During laparoscopic cholecystectomy, an accessory liver tissue attached to the gallbladder fundus by a pedicle was incidentally identified. The ectopic tissue was excised together with the gallbladder, and the postoperative course was uneventful. Although often asymptomatic, ectopic liver tissue carries a potential risk of malignant transformation. When encountered during cholecystectomy, it should be removed with meticulous attention to its vascular connection to avoid complications.

## Introduction

Tissues located outside the liver but anatomically connected to it are defined as accessory liver tissue, whereas those not anatomically connected to the liver are classified as ectopic liver tissue [1]. Ectopic liver tissue can be found in various intra-abdominal locations, including the retroperitoneum, stomach, spleen, and gallbladder [1-3]. The incidence of ectopic liver tissue diagnosed during laparotomy and laparoscopy ranges between 0.24% and 0.47% [4]. Among 5,500 autopsies reviewed, ectopic liver tissue was identified in 0.05% of cases, three of which were located on the gallbladder wall [5].

Although ectopic liver tissue is usually an asymptomatic developmental anomaly detected incidentally, it can become symptomatic and complicated under certain conditions, such as torsion or intra-abdominal bleeding, potentially requiring emergency surgical intervention. Gallbladder-associated ectopic liver tissue, in particular, may present with nonspecific biliary symptoms or mimic other gallbladder pathologies, making clinical recognition more challenging [1].

Another important complication is malignant transformation. Similar to the main liver, ectopic liver tissue can be infected by hepatitis viruses and may develop steatohepatitis and cirrhosis. Small ectopic livers lacking functional biliary architecture may be more exposed to chemical carcinogens secreted by hepatocytes, potentially making them more susceptible to carcinogenesis than the main liver. Although statistical analysis is difficult, definitive evidence for increased carcinogenic predisposition is lacking. However, 22 cases of hepatocellular carcinoma (HCC) in ectopic liver tissue, without involvement of the main liver, have been reported [3]. In this case, we aimed to present an instance of accessory liver tissue located on the gallbladder and to discuss its clinical significance in light of the current literature.

## Case presentation

**Figure 1:**
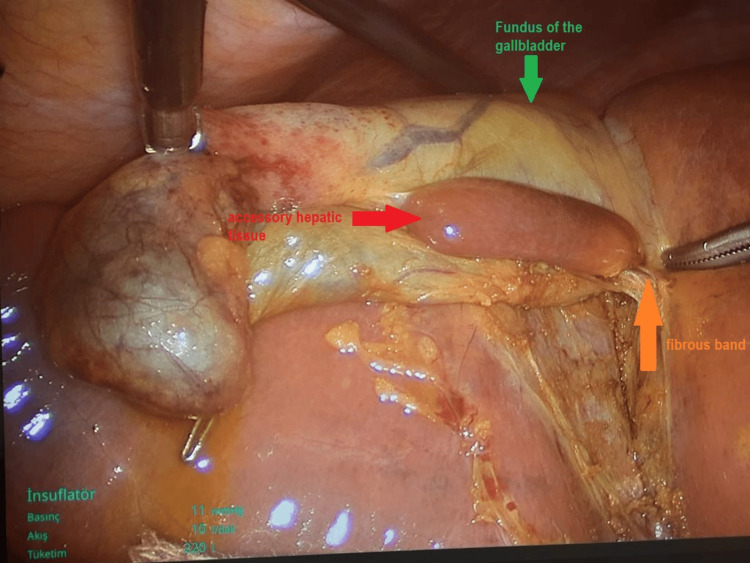
Accessory hepatic tissue was found at the fundus, linked to the main liver by a fibrous band.

A 58-year-old female patient with a 13-year history of type 2 diabetes mellitus and chronic steatohepatitis presented with three prior episodes of biliary colic; however, she had no active abdominal pain at the time of admission. Physical examination findings were unremarkable. Cholecystectomy was planned for biliary colic. Preoperative abdominal ultrasonography revealed a 1 cm gallstone, an enlarged liver (long axis 183 mm), and grade 2 steatosis. Preoperative liver function tests were within normal limits, including aspartate aminotransferase (AST) 13.9 U/L (normal: 10-40 U/L), alanine aminotransferase (ALT) 9.4 U/L (normal: 7-56 U/L), gamma-glutamyl transferase (GGT) 37.3 U/L (normal: 9-48 U/L), alkaline phosphatase (ALP) 89.5 U/L (normal: 44-147 U/L), total bilirubin 0.31 mg/dL (normal: 0.1-1.2 mg/dL), and direct bilirubin 0.13 mg/dL (normal: 0.0-0.3 mg/dL). Infection markers were also within normal ranges, with no leukocytosis or elevation in C-reactive protein. At the start of surgery, the gallbladder was lifted cranially, revealing accessory liver tissue connected to the main liver via a fibrous pedicle at the fundus (Figure 1).

**Figure 2:**
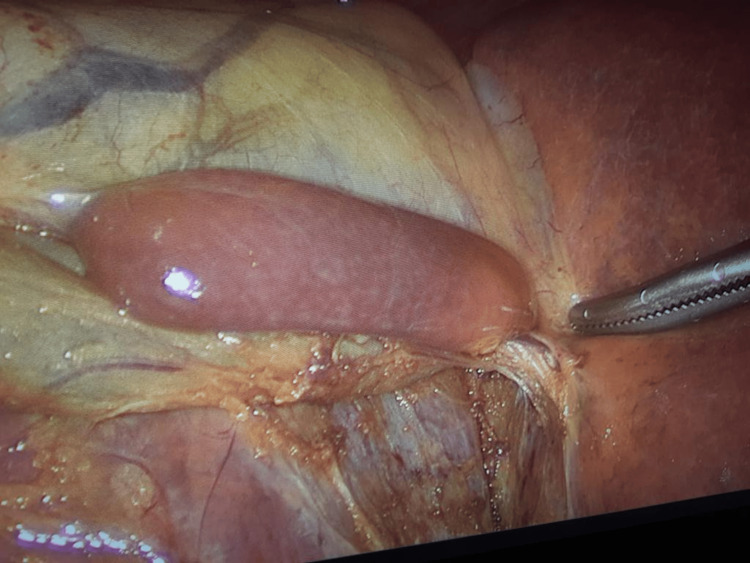
The pedicle was carefully retracted to avoid bleeding.

Dissection of Calot’s triangle identified normal cystic artery and cystic duct anatomy. Both were clipped and transected. During detachment of the gallbladder from the liver bed, gentle traction was applied to the accessory liver pedicle to prevent bleeding (Figure 2).

The connection was transected using a sealing device. No bleeding or bile leakage occurred. The accessory liver tissue was removed en bloc with the gallbladder using an endobag. A Jackson-Pratt drain was placed in the subhepatic space to monitor potential bile leakage [6]. After 24 h, 30 cc of serohemorrhagic fluid was noted, and the drain was removed. The patient was discharged the following day without complications. Macroscopic examination revealed a 7.5×3 cm gallbladder with a 2.7×1.2×0.7 cm nodular ectopic liver tissue on the fundus. Histopathology showed no hepatocellular carcinoma.

## Discussion

Ectopic liver tissue is a rare developmental anomaly in which hepatic parenchyma is located outside the main liver. Hepatic tissue located outside the liver is categorized into the following four types based on its anatomical connection and size: accessory liver of significant size and function connected via a pedicle; small accessory liver weighing 10-30 g also connected via a pedicle; ectopic liver tissue not anatomically connected, often found on the gallbladder; and microscopic liver tissue identified histopathologically from samples taken during surgery but not noticed intraoperatively [7,8]. Extrahepatic tissue is also classified into three types based on blood supply. Ectopic liver tissue may receive vascular supply via a small mesentery [9]. If the blood supply is through a pedicle containing vessels from the main liver parenchyma, it should be classified as an accessory liver [10]. Additionally, ectopic liver tissue can be supplied by a branch of the cystic artery [11].

Previous reports of gallbladder-associated ectopic liver tissue describe similar incidental findings during cholecystectomy, most commonly at the gallbladder fundus or serosal surface [1,12,13]. In published cases, the ectopic tissue typically presents as a small nodular mass attached by a pedicle or fibrous band, often with vascular supply derived from the cystic artery, consistent with the vascular pattern observed in our case [1,12,13]. Most reported patients are asymptomatic, with ectopic tissue discovered intraoperatively during surgery for biliary colic or cholelithiasis - again aligning with our patient’s clinical presentation [1,12]. Malignant transformation, although rare, has been documented in several case reports, underscoring the importance of complete excision when encountered [1]. Overall, the present case mirrors the typical clinical, anatomical, and operative features described in the literature, further contributing to the understanding of this uncommon entity [1,12,13].

Ectopic liver tissue is usually asymptomatic and incidentally diagnosed during laparotomy or laparoscopy. However, complicated ectopic livers may present with symptoms, such as torsion causing sudden, stabbing pain inconsistent with physical findings; intra-abdominal bleeding secondary to trauma; spontaneous bleeding due to rupture of malignant ectopic tissue; or hemorrhagic necrosis presenting with nonspecific symptoms like epigastric pain and nausea. These conditions require resection of the ectopic tissue, and if located on the gallbladder, cholecystectomy is performed [11].

Due to the risk of malignant transformation, incidental ectopic liver tissue should be excised [14]. In the literature, 22 cases have reported hepatocellular carcinoma (HCC) arising from ectopic liver tissue without involvement of the main liver [3]. Therefore, histopathological evaluation following excision is essential for confirming benignity and ruling out malignancy.

In our case, accessory liver tissue was found incidentally during cholecystectomy for biliary colic, with no evidence of malignant transformation in the pathological examination. Intraoperative differentiation between ectopic and accessory liver tissue is crucial, as accessory liver tissue possesses a pedicular connection containing vessels and bile ducts. During dissection, gentle traction should be applied to avoid bleeding or bile duct injury. If the presence of a bile duct within the pedicle is suspected, clipping before transection is recommended [3,11]. When ectopic liver tissue is found on the gallbladder, postoperative placement of a drain is advised, even if no intraoperative bile leakage is observed, as it facilitates early detection of potential complications.

## Conclusions

Ectopic and accessory liver tissues are typically asymptomatic and are most often discovered incidentally during surgical procedures. However, they may also present acutely with complications, such as torsion, spontaneous hemorrhage, hemorrhagic necrosis, or malignant transformation. When these tissues are identified on the gallbladder, standard cholecystectomy should be performed with meticulous attention to the vascular anatomy, and histopathological examination is essential to exclude hepatocellular carcinoma.
